# Adult-Onset Niemann–Pick Disease Type C: Rapid Treatment Initiation Advised but Early Diagnosis Remains Difficult

**DOI:** 10.3389/fneur.2017.00108

**Published:** 2017-04-04

**Authors:** Tobias Piroth, Kai Boelmans, Florian Amtage, Michel Rijntjes, Anna Wierciochin, Thomas Musacchio, Cornelius Weiller, Jens Volkmann, Stephan Klebe

**Affiliations:** ^1^Department of Neurology, University Hospital Freiburg, Freiburg im Breisgau, Germany; ^2^Department of Neurology, University Hospital Würzburg, Würzburg, Germany

**Keywords:** Niemann–Pick disease type C, adult-onset, NPC1 gene, NPC2 gene, plasma oxysterols

## Abstract

Niemann–Pick type C disease (NP-C) presents with heterogeneous neurological and psychiatric symptoms. Adult onset is rare and possibly underdiagnosed due to frequent lack of specific and obvious key symptoms. For both early and adolescent/adult onset, the available data from studies and case reports describe a positive effect of Miglustat (symptom relief or stabilization). However, due to the low frequency of NP-C, experience with this therapy is still limited. We describe two adult-onset cases of NP-C. In both cases, vertical supranuclear gaze palsy was not recognized at symptom onset. Correct diagnosis was delayed from onset of symptoms by more than 10 years. The video demonstrates the broad spectrum of symptoms in later stages of the disease. Compared with published data, the treatment outcome observed in our cases after delayed initiation of Miglustat therapy was disappointing, with continuing disease progression in both cases. Thus, early treatment initiation could be necessary to achieve a good symptomatic effect. Hence, early biochemical testing for NP-C should be considered in patients suffering from atypical neurological/neuropsychological and psychiatric symptoms, even in cases of uncertainty.

Niemann–Pick type C (NP-C) is a rare neurodegenerative lysosomal storage disorder of autosomal recessive inheritance. NP-C is caused by mutations of either the NPC1 (95% of families) (1) or the NPC2 gene (2). The underlying pathophysiology originates from impaired lipid trafficking, resulting in intracellular accumulation of unesterified cholesterol and other composites in various tissues, including the brain (3). The estimated incidence is 1/120,000–1/150,000 live births (3). The diagnosis of NP-C is established by a combination of genetic biochemical (oxysterols in plasma, filipin staining in fibroblasts) and genetic testing, which involves NPC1/2 gene sequencing (4). The age at onset (AAO) of most patients is from early infancy (<2 years) to adolescent/adult onset (≥15 years) (5). Adult onset is rare and patients present with a very heterogeneous clinical spectrum of symptoms such as cognitive impairment, cerebellar symptoms, dystonia, vertical supranuclear gaze palsy (VSGP), psychiatric disorders and, less frequently, epilepsy (6, 7). Due to the broad phenotypic spectrum, NP-C is easily overlooked in adult patients, in particular because VSGP might start as mild, isolated vertical saccade initiation (VSI) delay. As a consequence, time from first symptoms to NP-C diagnosis often takes several years. This delay is crucial, as early diagnosis and the initiation of an approved therapy with Miglustat (Zavesca^®^, Actelion Pharmaceuticals, Allschwil, Switzerland)—a reversible glycosphingolipid synthesis inhibitor that can stabilize or slow the progression of irreversible neurological damage—is of great importance (8). The recently improved Niemann–Pick type C Suspicion Index tool (NPC-SI) helps to detect patients that should be tested for NP-C (9). The test is available from http://www.npc-si.com/. We present the clinical course of two genetically confirmed NP-C cases with adolescent and adult AAO. Patient characteristics are provided in Table 1. Video S1 in supplementary material demonstrates clinical features of both patients after diagnosis.

**Table 1:** **Patient characteristics and imaging findings**.

	Patient 1	Patient 2
Sex	Female	Male
Age at presentation, years	28	48
AAO, years	17	38
NPC1 mutation	p.P474L/P474L	p.S954L/P1007A
First symptoms	Cognitive decline	Cognitive decline
Onset VSGP, years	33	?
Cognitive decline, years	17	38
Onset ataxia, years	23	?
MRI	Slight cerebral atrophy	Frontotemporal and cerebellar atrophy
FDG-PET	n.a.	Normal
NPC-SI at first admission	14 (revised version)	–
NPC-SI at diagnosis	76 (revised version)	76 (revised version)

*AAO, age at onset; FDG-PET, fluorodeoxyglucose positron emission tomography; MRI, magnetic resonance imaging; NPC-SI, Niemann–Pick type C Suspicion Index tool; VSGP, vertical supranuclear gaze palsy; ?, unknown; n.a., not applicable*.

Patient 1 (first part of the film) consulted a neurologist at the age of 28 years after experiencing frequent falls, ataxia, and mild mnestic deficits. Her parents reported a difficult, traumatic birth. Several developmental milestones (e.g., learning to walk or speak, etc.) during early childhood were achieved with a delay of several months. During that time, it was supposed that the condition comprised symptoms of infantile cerebral palsy, no hepato-, or splenomegaly had been observed. However, the clinical course during elementary and high school was unremarkable, and no developmental deficits were noted during that time. In the course of an apprenticeship at the age of 17 years, increasing difficulties using electrical tools forced her to quit her job. From then on, she was only capable of minor jobs and was finally employed in a protected workshop due to cognitive deficits. At the age of approximately 23 years, the patient developed a gait disorder that progressed over the following years.

During her first neurologic consultation 5 years later, clinical findings revealed slightly increased latency of VSI but no hypometric eye movements, no ataxia, and no obvious cognitive and psychomotor deficits. VSI was noted but was not interpreted as VSGP. Family history included death of one cousin due to a “severe neurologic condition,” which was never accurately diagnosed. Magnetic resonance imaging (MRI) revealed slight cerebral atrophy. Electroencephalographic recordings matched criteria for diffuse generalized encephalopathy. General cerebrospinal fluid (CSF) status was normal. The condition was interpreted as sequelae of the infantile cerebral palsy that had been presumed during childhood. NPC-SI at the time would have been 58 points (high likelihood of NP-C if VSGP was recognized) or 14 (low likelihood if VSGP was not recognized). During the following 3 years, symptoms progressed slowly. At the age of 33 years, hypometric vertical saccades were recognized as VSGP. An affection of the first motor neuron (spasticity, extensor plantar response) was noted. At the age of 34 years, the additional appearance of dystonic symptoms triggered another workup of this complex case and finally led to a genetic diagnosis of NP-C caused by a homozygous mutation of NPC1 gene (p.P474L/P474L). NPC-SI (revised version) was 76 points (high likelihood of NP-C).

Medical therapy with Miglustat 300 mg/day was initiated and has been used for more than 2.5 years at the time of writing this report. Due to continuing symptom progression, escalation of the dose up to 600 mg/day was attempted twice. However, worsening of ataxia reported by caregivers and appearance of tremor forced us to reduce the drug dosage. During the course of treatment, the patient became almost completely mutistic and gait ataxia has increased to such a level that she falls frequently and regularly requires assistance while walking. Twelve months after initiation of therapy, SARA score was 28/40 points and increased during the following 18 months to 32/40 points. Dysphagia (impaired initiation of swallows, but only rare coughing) and ataxia of the upper limbs cause dependence during many activities of daily living.

Patient 2 (second part of the film) first presented slow progressive concentration/memory impairment at the age of 38 years, followed by gait disturbances that worsened over time. No visceral symptoms (splenomegaly, prolonged unexplained jaundice) had been described in childhood. He had finished junior high school and undergone an apprenticeship to become a mechanic. Afterward, he worked as a train conductor until the age of 39 years when he was retired prematurely due to his cognitive deficits. At the age of 48 years, a frontotemporal degeneration (FTLD) was suspected because of a slight frontotemporal and cerebellar pronounced atrophy on the MRI. A fluorodeoxyglucose positron emission tomography (FDG-PET scan) at that time was normal. In the CSF, the tau (592 pg/ml; ref. <450 pg/ml) and phospho-tau (81 pg/ml; ref. <61 pg/ml) protein were slightly increased. The clinical examination revealed a cerebellar ataxia (SARA score 10/40), dysarthria, VSGP, and myocloniform movements in the proximal muscles. Neuropsychological testing showed deficits in visuo-constructive skills, working memory, and attention memory. The NPC-SI was 76 points (revised NPC-SI, high suspicion for NP-C). Due to the phenotype, diagnostic testing for NP-C was initiated, revealing increased oxysterols [0.11 ng/μl (<0.05)] in the serum and subsequently a compound heterozygous mutation in the NPC1 gene (p.S954L/P1007A). Therapy with Miglustat (3 × 200 mg) was started at 49 years of age. After 6 months of treatment, symptoms worsened due to development of frequent falls and apathy. Unfortunately, the patient died as a result of bolus aspiration at age 50.

Previous studies have shown that NP-C patients with juvenile or adult-onset phenotypes show even better responses to treatment than patients with an early onset in terms of improvement or stabilization of neurological symptoms (10). The most important outcome parameter to avoid worsening of neurological symptoms was the delay between the onset of symptoms and the initiation of treatment (10). The present cases of genetically confirmed NP-C cases exemplarily illustrate the serious dilemma of the latency in diagnosis of NP-C (17 years in patient 1; 10 years in patient 2). The Miglustat treatment that was initiated after the diagnosis was made was unfortunately only of little success.

The major reason for the delayed diagnosis in our two patients was due to the phenotypical difference between the early- and late-onset NP-C patients. Hepatosplenomegaly and/or gelastic cataplexy are less frequent or missed by the lack of an appropriate examination in patients with an adult onset. Other neurological manifestations (VSGP, cerebellar ataxia, dystonia, and dysarthria/dysphagia) are more frequent (11), in our patients even a key clinical sign of NP-C, the VSGP, only became apparent later in the disease course or was not correctly recognized early in the disease course. Systematic studies illustrate the broad spectrum of initial symptoms in adolescent- or adult-onset NP-C cases: in a report on five cases where disease onset was between 5 and 50 years of age, ataxia and myoclonus appeared to be the most frequent neurologic symptoms, while VSGP was lacking in two patients. Koens and coauthors described movement disorders as primary symptom in three-quarters of patients, mainly myoclonus (including negative myoclonus) and ataxia. Psychosis was less frequent, while cognitive deficits appeared early in the course of the disease in every second patient of the cohort (11).

Brain MRI or FDG-PET imaging might be normal or unspecifically changed in NP-C (6, 7), whereas specific changes have been described for neurodegenerative diseases such as progressive supranuclear palsy (PSP) or multiple system atrophy. In the past, lack of specific features in early stages of adult-onset NP-C led to a low or moderate NPC-SI score, as shown by others (6). On the other hand, the NPC-SI shows a high probability if it is applied in cases with clinical diagnoses of neurodegenerative diseases such as PSP or FTLD. However, a recent study in 790 patients suffering from PD, FTLD, or PSP and 846 age-matched controls showed comparable rates of NPC1/2 frequency variants for PD patients (1.1%) and controls (0.8%), and no polymorphism in FTLD and PSP patients (12). A recent study reported that NP-C could be identified even when patient selection is restricted to early onset cerebellar ataxia (13). In our view, metabolic NP-C testing should be offered to patients with a pre-senile cognitive decline with or without ataxia, VSGP with or without presentation of movement disorders, or psychiatric signs. The revised version of the NPC-SI supports screening of older cases, since it offers separate versions for patients below and above 4 years of age.

The finding of elevated levels of tau and phospho-tau in the CSF of patient 2 is particularly interesting. Gene expression data from fibroblasts of NPC patients suggest similarities to Alzheimer’s disease, where hyperphosphorylation of microtubule-binding protein tau reduces the microtubule-binding capability of tau and is associated with the formation of neurofibrillary tangles. In fibroblast derived from NPC patients, genes of proteins involved in tau phosphorylation are overexpressed in comparison to control cells (e.g., TTBK1, MAPK8, and PRKCG) (14). Studies in rodents have demonstrated increase in phenotype severity in NPC1/tau double null mutants, but not in tau knockout animals that carry the NPC1 wild-type gene (15). It also has been shown that tau reduction in NPC1-deficient fibroblasts—in contrast to control cells—decreases autophagy induction and flux (16). Systematic CSF analysis for tau phosphorylation levels might contribute to understanding of disease mechanisms and heterogeneity of clinical cases.

In both cases in the present report, Miglustat therapy was initiated after diagnosis of NP-C. However, the disease was not correctly diagnosed initially, causing a delay between onset of symptoms of 10 and 12 years, respectively. Unfortunately, we could not find symptomatic relief or at least stabilization. The first patient developed increasing impairment of mobility, ataxia, and dysarthria. Similarly, the second patient had increasing difficulties in walking. A potential life-threatening symptom of NP-C, neurogenic dysphagia, was not reduced in both patients and probably resulted in the death of the second patient.

Systematic reports describe a robust trend toward positive outcomes for Miglustat treatment in adult/adolescent-onset patients. Results from the most comprehensive NP-C registry to date included data from 18 adolescent/adult-onset patients receiving Miglustat (14). Disease severity is illustrated by a disease-specific disability score. The score is composed of subratings for ambulation, manipulation, language, and swallowing values, ranging from 0 (no impairment) to 1 (worst). The mean age of first symptoms in adult-onset patients was 22.7, while mean age at initiation of treatment was 26.7. The overall disability score during the observational period was stable and improved slightly from 0.35 (SD: 0.17) to 0.31 (SD: 0.22) (17). In a recent report, out of three patients that had received Miglustat continuously, one patient improved and two remained on a stable clinical level. In one patient within the same study, treatment was initiated and then withdrawn after severe digestive side effects. This patient died due to sudden-onset swallowing difficulties (6).

Several factors might contribute to the discrepancy between the overall positive outcome in recent publications and the experiences described in the present report. Patient 1 from the present series tolerated only a dosage of 300 mg daily, since she and her caregivers reported a worsening in disease severity immediately after increase to 600 mg, even if the dosage was increased in 100 mg steps every second week. For patient 2, the follow-up period is probably too short to allow for observation of a robust clinical improvement. He died only 12 months after treatment initiation. However, one would have expected at least some kind of stabilization in both cases given the overall positive results of Miglustat therapy that have been published recently. The limited response might be due to the delayed initiation of treatment and lower dosage in one of the two cases.

In conclusion, our case reports support the notion of a heterogeneous phenotype and disease course of NP-C. The frequency of NP-C testing in routine diagnostics should increase as disease biomarkers such as plasma oxysterols, even if not specific for NP-C, have recently become more easily available. Miglustat has been shown to be effective in infantile and adolescent-onset patients, but further research is warranted in order to understand heterogeneous outcomes.

## Ethics Statement

We confirm that we have read the Journal’s position on issues involved in ethical publication and affirm that this work is consistent with those guidelines.

## Author Contributions

TP conducted follow-up visits of patient 1 and recorded video sequences. He contributed to the development of the concept. KB and TM conducted follow-up visits of patient 2 and contributed to the clinical description. FA and MR conducted follow-up visits of patient 1 and contributed to the clinical description. AW participated in acquisition of data in patient 2. CW and JV critically revised the manuscript for important intellectual property. SK conducted and supervised follow-up visits of patients 1 and 2 and recorded video sequences. He developed the concept.

## Conflict of Interest Statement

Financial disclosures for the previous 12 months: TP has received travel funding from Actelion. KB has received travel compensation from Bayer Healthcare. FA has received travel grants from UCB Pharma and Ipsen Pharma. TM has received travel funding from AbbVie and Medtronic. JV has received research support from Medtronic and Boston Scientific and royalties from Medtronic, Boston Scientific, St. Jude, TEVA, UCB, AbbVie, and Allergan. SK has received travel funding from UCB and AbbVie and royalties from Actelion, UCB, and Thieme. The reviewer JPP and handling Editor declared their shared affiliation, and the handling Editor states that the process nevertheless met the standards of a fair and objective review.

## Supplementary Material

Video S1 **Illustration of motor symptoms in two adolescent/adult-onset NP-C patients**. 00:00: patient 1, approximately 15 years after onset of motor symptoms. 00:05: demonstration of supranuclear, predominantly vertical gaze palsy. Inhibition of horizontal saccade initiation and pursuit movements should also be noted. 00:35: upper limb ataxia. 00:51: lower limb ataxia. 01:09: gait ataxia; disabling limb dystonia is obvious. 01:35: NP-C patient 2. 01:40: vertical gaze palsy. 01:56: positive applause sign. 02:08: gait ataxia. 02:13: postural reflexes.
